# Clinical study of skin changes in low and high risk pregnant women*

**DOI:** 10.1590/abd1806-4841.20153570

**Published:** 2015

**Authors:** Lana Bezerra Fernandes, Waldemar Naves do Amaral

**Affiliations:** 1 Universidade Federal de Goiás (UFG) - Goiânia (GO), Brazil

**Keywords:** Pemphigoid gestationis, Pregnancy, Skin, Skin diseases, Skin pigmentation

## Abstract

**BACKGROUND:**

During pregnancy there is immunological, metabolic, endocrine and vascular changes responsible for physiological and pathological skin changes.

**OBJECTIVES:**

determine the prevalence of specific physiological changes and pregnancy, comparing the period of gestation of their appearances and compare type of prenatal care as the skin changes.

**METHODS:**

A cross-sectional study with 905 pregnant women.

**RESULTS:**

The prevalence of physiological skin changes was 88.95% and the most common was pigment. The prevalence of specific dermatoses was 8.72% and atopic eruption was the most common.

**CONCLUSION:**

Physiological changes were seen more in the 3rd quarter, as well as the specific dermatoses. No statistical difference in prenatal low risk compared to high risk was observed, whereas the cutaneous physiological changes and specific pregnancy dermatoses.

## INTRODUCTION

Pregnancy is a period of several changes for women. Practically all body systems are affected. And among the systems affected during pregnancy is the skin. Most changes in the female body are mechanical and/or hormonal. These are characterized by high elevations of estrogen, progesterone, beta-HCG (chorionic gonadotropin), prolactin and a variety of hormones and mediators that completely alter the body's functions.^1^ Regarding the skin, gestational changes are divided into: 1) gestational physiological changes, 2) specific dermatoses and 3) dermatoses changed during pregnancy.^1,2^

Most recent classification was proposed by Ambros-Rudolph et al in 2006, after a retrospective study in two centers with 505 pregnant women. Among pregnancy specific changes are: 1) gestational pemphigoid (GP), 2) polymorphic eruption of pregnancy (PEP), 3) atopic eruption of pregnancy (AEP) and 4) intrahepatic cholestasis (IC).^2^

Although rare, specific dermatoses of pregnancy, such as gestational pemphigoid and intrahepatic cholestasis, have an increased risk of adverse events on the fetus, and it is important to distinguish them from physiological changes and previous dermatoses changed during pregnancy - baseline diseases that simply present or worsen during pregnancy.^3^

## METHODS

This is a cross-sectional, analytical and quantitative study conducted from May 2011 to April 2013. Sample size was calculated using finite populations (n=3,000 new cases from the clinic). It was set up a significance level of 5%, a percentage P = 50% (implying maximum sample size) and a sampling error of 3.6%, with the minimum number of assessed pregnant women of 600. This study was conducted in a population of pregnant women assisted in high and low risk prenatal clinics of Hospital das Clínicas da Universidade Federal de Goiás (HC-UFG). Patients were submitted to only a dermatological assessment in the same day of the first prenatal examination in the service; 905 pregnant women were evaluated only one time for the analysis to have a better sample distribution as to quarters of pregnancy under study. Pregnant women referred from the first quarter or only in the third quarter were evaluated only once by a dermatologist, and in a cross-sectional and prevalence study. Skin, hair, mucous membranes and nails were assessed, following a head-to-toe examination dynamic in optimal light conditions. Data were collected following an order present in this survey questionnaire, and a rigorous physical examination was conducted. Laboratory tests and biopsies for differential diagnoses were requested. Variables were studied through relative and absolute frequency calculations and chi-square test for categorical variables. It was set a value of 5% for significance.

## RESULTS

A total of 905 pregnant women was evaluated in this study: 805 of them presented physiological changes and 79 presented specific dermatoses.

### Physiological changes

Among the 905 evaluated pregnant women, 805 (88.95%) presented physiological changes; of these, 708 (87.95%) had hyperpigmentation, such as emergence of linea nigra, increased pigmentation of mucous, melasma and increased melanocytic nevus; 425 (46.96%) had formation of new stretch marks; and 373 (41.21%) presented vascular changes (Table 1). Among pregnant women with physiological changes, 284 (35.27%) were primiparous and 521 (64.72%) were multiparous. Pregnant women's ages ranged from 15 to 45 years with a mean of 26.32 years. Most of them presented high skin type (IV-V): 362 (44.96%).

**Table 1:** Distribution of pregnant women with physiological skin changes, assisted at the Obstetrics Clinic/HC/ UFG from May 2011 to April 2013

Physiological changes		Number of patients (N=805)	
		N	%
**Pigmentation**			
	Linea Nigra	495	54.75
	Melasma	489	54.03
	Increased nevus	375	41.43
**Stretch marks**		425	46.96
**Vascular**		373	41.21
**Glandular**		324	35.80
Hair changes			
	Increased growth and volume	190	20.99
	Increased hair loss	100	11.04
**Nail changes**		73	8.06

In the analysis of the occurrence of physiological changes, a greater gestational age (quarter) was related to a greater prevalence of physiological changes. In the first quarter, physiological changes were observed in 13.04% of the total cases; in the second quarter, in 19.88%, and in the third quarter, in 56.02%; thus presenting a statistical difference among quarters and a higher prevalence in the last quarter. When comparing the emergence of physiological changes with the type of prenatal (low and high risk), this difference was not significant (p=0.505) (Table 2).

**Table 2:** Distribution of patients with physiological changes according to gestational age of involvement in quarters and type of prenatal care, assisted in the Obstetrics Clinic from May 2011 to April 2013

	Quarter							
Type of prenatal carea	1st		2nd		3rd		P
	N	%	N	%	N	%	
Low-risk	65	55.1	93	51.7	250	49.3	
High-risk	53	44.9	87	48.3	257	50.7	0.505
Total	118*	100	180*	100	507*	100	
	(13,04%)		(19.88%)		(56.09%)		

Chi-square test;

* Between trimestres: p<0.001

### Specific dermatoses

Of the total of 905 pregnant women evaluated, 79 (8.72%) showed specific dermatoses. Atopic eruption was the most common specific dermatoses: 56 women (70.88%); followed by intrahepatic cholestasis, 15 (18.98%) and polymorphic eruption of pregnancy, 8 (10.12%) (Table 3). No case of gestational pemphigoid was observed. Of the total of 79 pregnant women, 29 (36.70%) were primiparous and 50 (63.29%) were multiparous. Pregnant women's ages ranged from 22 to 39 years, with a mean of 30.5 years. Most of them presented high skin type (IV-V), 32 (40.50%).

**Table 3:** Distribution of pregnant women with specific dermatoses assisted at Obstetrics Clinic/ HC/ UFG from May 2011 to April 2013

Specific changes	Number of patients (N=79)	
	n	%
Atopic eruption	56	70.88
Intrahepatic cholestasis	15	18.98
Polymorphic eruption	8	10.12

In the analysis of the occurrence of specific dermatoses, the third quarter was the only one to present statistical difference compared with the first and second quarters of pregnancy. When comparing the emergence of specific dermatoses with the type of prenatal (low and high risk) this difference was not significant (Table 4). Distribution of pregnancy specific dermatoses varied according to gestational age into quarters, with a higher prevalence in the third quarter, especially in cases of intrahepatic cholestasis of pregnancy and polymorphic eruption of pregnancy, mainly related to weight gain and twin pregnancy. Moreover, atopic eruption of pregnancy was observed in all the quarters, being most evident in the second and third quarters.

**Table 4:** Distribution of pregnant women with specific dermatoses according to gestational age (quarters) and type of prenatal (low and high risk), assisted at Obstetrics Clinic/ HC/ UFG from May 2011 to April 2013

Type of prenatal	Quarter (N=79)						p (risk)
	1st		2nd		3rd		
	N	%	N	%	N	%	
Low-risk	5	35.71	12	60.00	24	53.33	0.362
High-risk	9	64.29	8	40.00	21	46.67	
Total*	14^A^	100	20^B^	100	45^A,B,C^	100	

## DISCUSSION

Pregnancy causes profound organic changes in women, making them sensitive to skin changes and attachments that can be physiological or pathological.^4^ Conflicting and overlapping classifications on specific dermatoses of pregnancy contributed to the diagnostic confusion and difficulty in establishing prevalence.^2^

Pregnant women with physiological changes had mean age of 26.32 years and specific dermatoses of 30.5 years. These data are consistent with data obtained in Rathore and Gupta (2011) study, in which the mean age of physiological changes was 26.42 years in 2000 pregnant women evaluated. According to Ambros-Rudolph et al (2006), the mean age of specific dermatoses was also 30 years in 505 pregnant women evaluated.^2,5^ There wasn't, therefore, statistical difference in mean age between women with and without gestational dermatoses in our study.

In the Midwest, most pregnant women is skin type IV-V (40%), given the Indian and black influences in this population and the presence of patients from North and Northeast regions. In Goiás, 50% of the population is self-reported as brown and 6.5% as black.

Analyzing the final diagnosis of skin changes in patients, the presence of physiological changes was 88.95%, according to studies carried out in India by Kumari, Jaisankar and Thappa (2007), with 607 pregnant women, and by Muzaffar et al (1998), with 140 pregnant women, in which 100% of patients studied presented physiological changes during pregnancy.^4,6^

Among physiological changes found in our study, hyperchromia were present in 87.95% of pregnant women. Pigmentary changes are extremely common, affecting up to 90% of pregnant women.^4,6^ Prevalence of melasma was 54.03%, a result similar to studies by Wong and Ellis (50-70%) ^7^ and by Muzaffar et al (46.4%).^4^

New stretch marks were observed in 425 pregnant women (46.96%), and in 360 of them (84.70%) the marks emerged in the third quarter, mainly in the lower abdomen and breasts, especially due to the weight gain and increased abdominal volume (Osman et al, 2007); ^8^ and 276 (64.94%) of these patients were primiparous, a result similar to that from the study of Muzaffar et al (1998).^4^

Vascular changes, including appearance of spider veins, varicosities, hemangioma and granuloma, for example, were observed in our study in 41.15% of cases, with results similar to those from Schumtz study (2003), of 50-70% cases.^9^

There was a predominance of skin physiological changes in pregnancy in the third quarter (56.02%), with similar results compared with studies by Wong and Ellis (1984) ^7^ and by Muzaffar et al (1998).^4^ There was no statistical difference between low and high risk prenatal, because these lesions emerge as an expected natural event and do not show correlation with comorbidities present in high-risk prenatal care. No study observed significant comorbidities in skin physiological changes of pregnancy.

Regarding specific dermatoses of pregnancy, in this study we used the most common classification in the literature, proposed by Ambros-Rudolph et al, in 2006.^2^ It was observed that among patients treated in the Obstetrics Clinic, of a total of 905 pregnant women evaluated, 79 (8.72%) presented specific dermatoses. However, the incidence of specific dermatoses is 0.5% to 3%, according to studies by Roger and Vaillant (1994).^10^ The increased prevalence of specific dermatoses may be justified, to some extent, by the great incidence of high-risk pregnancies with comorbidities, such as twin pregnancy, obesity and atopies present in the clinic; and the differences can be explained in part by different classifications adopted in each study. The current classification of atopic eruption of pregnancy encompasses other classifications and types of dermatoses before subdivided, thus leading to an increased prevalence in this population.

Atopic eruption was the most common specific dermatoses, with 56 cases (70.88%), followed by intrahepatic cholestasis, with 15 (18.98%), and polymorphic eruption of pregnancy, with 8 (10.12%). What contributed to the prevalence of atopic eruption (70.88%) in this study was the fact that this is a dermatoses that encompasses eczemas, prurigo of pregnancy and pruritic folliculitis, according to the newest classification proposed by Ambros-Rudolph, with similar results compared with studies by Rashmi and Devinder and Ambros-Rudolph et al, published in 2006.^2,11^ Moreover, the results differ from studies by Ram and Taru, in which there was a higher prevalence of intrahepatic cholestasis; and the prevalence of specific dermatoses was 5% among 1430 pregnant women, suggesting therefore a significant difference with our study.^11^

Our study observed a prevalence of atopic eruption in the three quarters and also an association with atopic dermatitis in 62% cases, with a higher prevalence in the third quarter, which differed from Ambros-Rudolph et al study (2006), in which there was a predominance of atopic eruption of pregnancy in the first quarters (75% of pregnant women).^2^ Also, 20% of patients had exacerbation of a preexisting atopic dermatitis and 80% presented the condition for the first time during pregnancy. Prevalence of polymorphic eruption and intrahepatic cholestasis occurred most in the last quarters, mainly in the third quarter, associated to overweight in most cases. Atopic eruption occurred early, while polymorphic eruption of pregnancy, gestational pemphigoid and intrahepatic cholestasis were presented later.^2^ In this study, there was a higher prevalence of skin diseases in the third quarter, in particular atopic eruption of pregnancy, probably due to the greater number of cases of worsening of atopic dermatitis (62%) and overweight. There was no statistical difference when comparing the type of prenatal.^12^

Despite all these considerations, skin changes in pregnant women are often neglected or ignored by health professionals, especially in pregnant women with physiological skin changes. Thus, appropriate multidisciplinary monitoring of pregnant women has important significance in screening and in clinical diagnosis of many systemic diseases with repercussions on the skin, as well as to identify risk factors of the pregnant woman and the fetus, being an important tool for prognosis and specific therapeutic indication of skin disorders during pregnancy.

We hope this study will contribute to draw attention of health professional to the need for educational investment for skin disorders that, despite low morbidity, present a high prevalence and a great discomfort. Results showed the importance of including a skin care approach in educational programs in health services to improve the care of pregnant women.

## CONCLUSION

Prevalence of cutaneous physiological changes during pregnancy was 88.95%, and specific dermatoses of pregnancy was 8.72%. The most common time of onset of physiological changes was the third quarter, as well as of specific dermatosis. There was no statistical difference in low risk compared with high risk prenatal, considering skin physiological changes and specific dermatoses.
